# Chemical Safety Studies: Conrad and Becker Respond

**DOI:** 10.1289/ehp.1104385R

**Published:** 2011-12-01

**Authors:** James W. Conrad, Richard A. Becker

**Affiliations:** Conrad Law & Policy Counsel, Washington, DC, E-mail: jamie@conradcounsel.com; American Chemistry Council, Washington, DC

Sutton et al. and Tweedale both criticize our review (Conrad and Becker 2011) because we did not include industry funding of a study in our proposed set of criteria. Fundamentally, we rejected “industry funding” as a credibility criterion “because questions can also arise about the credibility of research by scientists funded by government agencies or nonprofit organizations” (Conrad and Becker 2011). We accept as a given that someone has always paid for scientific work and that funding has inherent potential to influence results, whether it comes from industry, environmental groups, or government. It appears that at least one member of Sutton et al. agrees with us to some degree on this point, because they repeatedly cite Bero (1999), who endorsed “establishing restrictions on sponsorship, regardless of its source.”

We agree that funding bias has been documented, at least in clinical trials for pharmaceuticals and medical procedures, although as Sutton et al. note, the published literature does not yet appear to have systematically studied the issue in the field of toxicology or epidemiology across a broad spectrum of substances. (Tobacco is a unique and extreme case and should be recognized as such, not cited tendentiously as indicative of all industry support of research.) The guidelines for routine toxicity studies are publically available and incorporate end points reflecting both input from a broad spectrum of experts and approval by government regulatory authorities [U.S. Environmental Protection Agency (EPA) 2011], and all such studies employ an entirely independent quality assurance program documenting that facilities, equipment, personnel, methods, practices, records, and controls are in conformance with Good Laboratory Practice (GLP) requirements. The extent to which these features govern the design and conduct of toxicity studies mitigates funding bias to a large degree.

In our review (Conrad and Becker 2011), we also noted that source of funding is often considered a “less significant” cause of publication bias than other causes [e.g., career advancement (publish or perish), personal advancement, status in one’s professional field, interest in obtaining positive results].

Sutton et al. attempt to show that the documents from ostensibly nonindustry-funded sources that we cited in our review (Conrad and Becker 2011) in fact diverge from the other source documents, but their effort is unconvincing. First, they quote the Bipartisan Policy Center’s statement that one should “consider sources of funding and any conflicts of interest” associated with a study (Bipartisan Policy Center 2009). But that is consistent with our review. We did not urge readers to dismiss questions of funding; rather, we stated that such questions “trigger application of the criteria” (Conrad and Becker 2011). The statement Sutton et al. quote from the Federation of American Societies for Experimental Biology (FASEB; Brockway and Furcht 2006) is taken somewhat out of context. Brockway and Furcht (2006) pointed out that human-subjects research is a case of special concern, and they also addressed who should be permitted to participate in conducting research. But their larger goal was as we characterized it: given that “academia-industry collaborations can benefit society,” they proposed “voluntary measures that guard against research bias and foster transparency and accountability” (Brockway and Furcht 2006).

It is unclear what Sutton et al.’s solution—“application of systematic and transparent methodologies to vet the science”—would mean in practice when one is confronted with a toxicological or epidemiological study. We suspect that their solution would end up looking a lot like our criteria; for example, Bero (1999) noted the danger of suppression by sponsors, but our criteria 2, 3, and 7 (Conrad and Becker 2011) all militate against that possibility. Bero (1999) also argued that “sponsored investigators should retain control over the publication of results, regardless of their outcome”—our criterion 2. Tweedale likewise endorses “the simplicity and finality of forbidding outsider control of a researcher’s data,” but again, that is what a sponsor has to accept to satisfy criterion 2.

Sutton et al. complain that our criteria would not “eliminate” bias, but we do not claim to do that. We claim that each of our criteria “either *a*) increases confidence that the sponsor or experimenter did not shape or skew the results or interpretation of an experiment; or *b*) enables others to assess independently whether such shaping or skewing occurred.” Our criteria allow the scientific evidence to speak for itself.

Tweedale criticizes our review (Conrad and Becker 2011) for not proposing “financial conflict of interests [as] the lead criteria to assess data quality” and for “fail[ing] to mention” the funding bias issue. As to the former, the purpose of our review was to address credibility, not reliability; this is important because generally accepted methods for determining data reliability have already been adopted and implemented by regulatory agencies (European Chemicals Agency 2009; U.S. EPA 1999). As to the latter, in our review (Conrad and Becker 2011) we stated that “critics have argued that industry-supported work has employed methods, animal strains, or other test features that tend to miss or underestimate adverse effects,” so we clearly acknowledged the underlying concern, even if we did not cite Tweedale’s references.

Beyond conflict of interest, Tweedale additionally mischaracterizes our review (Conrad and Becker 2011) regarding the topic of GLP. We did not propose excluding any relevant study simply because it did not follow GLP. Consistent with established best practices of systematic evidence-based reviews, we support use of transparent, objective criteria for determining data quality and study reliability. Such criteria allow data from laboratory experiments, epidemiological investigations, and cutting-edge mechanistic research from all relevant studies, GLP and non-GLP, and from all investigators, regardless of affiliation or funding source, to be comprehensively and systematically reviewed, given appropriate weight, and integrated in a manner that provides a robust understanding of the mode of action and the potential hazards and risks that exposures to a substance could pose. These basic principles of causal inference are widely endorsed and practiced (e.g., National Research Council 2011), and such analysis will reveal the strengths and flaws of a study, independent of study authorship or funding.

Tweedale ignores or misunderstands *a*) previous discourse (Becker et al. 2009, 2010; Tyl 2009) explaining how and why the elements of GLP often result in greater weight being given to such studies, and *b*) the processes by which the Organisation for Economic Co-operation and Development (OECD) develops test guidelines, in which experts around the world collaborate to formulate, validate, update, and independently peer review OECD test guidelines (e.g., OECD 2008). When new end points or metrics can be shown to be valid, relevant, and reliable for assessing hazard and risk, they can be and are incorporated into new and revised OECD test guidelines. In the meantime, nothing prohibits Tweedale or “independent, curious academics” from providing a full study report and all raw data from their studies to regulatory agencies, as is routinely done for GLP studies, especially given that supplying underlying data will likely be a future requirement of journals (see Hanson et al. 2011).
